# The key molecular pattern BxCDP1 of *Bursaphelenchus xylophilus* induces plant immunity and enhances plant defense response *via* two small peptide regions

**DOI:** 10.3389/fpls.2022.937473

**Published:** 2022-08-03

**Authors:** Long-Jiao Hu, Xiao-Qin Wu, Tong-Yue Wen, Jian-Ren Ye, Yi-Jun Qiu, Lin Rui, Yan Zhang

**Affiliations:** ^1^Co-Innovation Center for Sustainable Forestry in Southern China, College of Forestry, Nanjing Forestry University, Nanjing, China; ^2^Institute of Botany, Jiangsu Province and Chinese Academy of Sciences, Nanjing, China

**Keywords:** *Bursaphelenchus xylophilus*, *Pinus thunbergii*, key peptides, immunity induction, protein interaction

## Abstract

The migratory plant-parasitic nematode *Bursaphelenchus xylophilus* is the pathogen of the pine wilt disease (PWD), causing serious damage to pine forests in China. During the process of plant resistance to multiple pathogens, plant immunity plays a key role. In this current study, the pathogen-associated molecular pattern (PAMP) BxCDP1 in *B. xylophilus* has been identified, but the host target protein of BxCDP1 and its key amino acid region inducing the plant immunity have yet to be elucidated. We found that BxCDP1 could trigger superoxide production, H_2_O_2_ production, and callose deposits. A RING-H2 finger protein 1 (RHF1) of *Pinus thunbergii* was screened and characterized as a target protein of BxCDP1 by yeast two-hybrid and co-immunoprecipitation (Co-IP). Moreover, two peptides (namely M9 and M16) proved to be key regions of BxCDP1 to induce PAMP-triggered immunity (PTI) in *Nicotiana benthamiana*, which also induced the expression of pathogenesis-related (PR) genes (*PtPR-3*, *PtPR-4*, and *PtPR-5*) in *P. thunbergii* and enhanced the resistance of the host to *B. xylophilus*. These results indicate that BxCDP1 plays a critical role in the interaction between *B. xylophilus* and *P. thunbergii*, and both peptides M9 and M16 have the potential to be developed and utilized as immune inducers of pines against *B. xylophilus* in future.

## Introduction

The pine wood nematode (PWN) *Bursaphelenchus xylophilus*, as an important migratory plant-parasitic nematode (PPN), causes the pine wilt disease (PWD). At present, PWD has become the most serious pine forest disease in China (Zhou et al., 2017; Huang L. et al., 2019; Wang et al., 2022). Due to the complexity of the occurrence and development of PWD, the exact pathogenic mechanism of *B. xylophilus* is not completely clear, making PWD difficult to predict and manage.

During the interaction of pathogens and plants, plants evolve a sophisticated basal immune system, and pathogens must overcome or escape plant immunity to colonize (Jones and Dangl, 2006). GPlants have two layers of the innate immune system. In the first tier, pathogen or microbe-associated molecular patterns (PAMPs or MAMPs, respectively) are recognized by plant plasma membrane-bound receptors (pattern recognition receptors [PRRs]) to induce innate immunity (PAMP-triggered immunity [PTI]) (Yang et al., 2017). In turn, the pathogen secretes effectors to suppress PTI in order to promote infection. In the second tier, plants employ the nucleotide-binding and leucine-rich repeat (NB-LRR) proteins encoded by disease resistance (R) genes to recognize effectors and trigger further innate immunity (effector-triggered immunity [ETI]) (Chisholm et al., 2006; Jones and Dangl, 2006). Many studies showed that PAMPs and effectors of pathogens have a key role in the interaction of pathogens and plants, which have become research hotspots (Jing et al., 2016; Chen et al., 2017; Kettles et al., 2017; Du et al., 2021).

Nowadays, research on PAMPs and effectors of *B. xylophilus* has attracted much attention. For example, effectors were broadly predicted and screened by transcriptomic analysis (Espada et al., 2016; Tsai et al., 2016; Margarida et al., 2018; Tanaka et al., 2019; Hu et al., 2021). Effectors BxSapB1, BxSapB2, and BxSapB3 were shown to trigger cell death and contribute to virulence (Hu et al., 2019; Huang X. et al., 2019; Zhao et al., 2020). A PAMP BxCDP1 was characterized in *B. xylophilus* (Hu et al., 2020). An effector Bx-FAR-1 inhibits plant immunity by mediating the jasmonic acid pathway in pines (Li et al., 2020; Wen et al., 2022). An effector BxSCD1 suppresses PAMP BxCDP1-triggered immunity and interacts with a host target protein PtACO1 (Wen et al., 2021).

In nature, oxygen molecules are in a less active oxidation state. However, when plants are subjected to biological stress such as pathogen infection, the dynamic balance of electron transfer and redox in plants is destroyed, and the electrons generated in cells can easily convert oxidized oxygen into reduced oxygen, producing superoxide [containing superoxide anion (O^2–^), hydroxyl free radical (OH^–^), and hydrogen peroxide (H_2_O_2_)]. These substances are collectively referred to as reactive oxygen species (ROS) (Wojtaszek, 1997). Plant immunity is stimulated by the accumulation of ROS, deposition of callose, induction of pathogenesis-related (PR) genes, and regulation of hormone signaling (Dodds and Rathjen, 2010; Tsuda and Katagiri, 2010). In our previous study, a PAMP of *B. xylophilus* BxCDP1 was identified, which triggered cell death and plant immunity in *Nicotiana benthamiana* and enhanced defense responses of *P. thunbergii* (Hu et al., 2020). However, it remains unclear whether BxCDP1 can activate the burst of ROS by increasing the content of superoxide and H_2_O_2_ and whether it can improve the deposition of callose in the plant.

The function of a protein is usually determined by its amino acid composition and protein structure (Li et al., 2018). The key amino acid regions of triggered or inhibited plant immunity of several PAMPs or effectors have been characterized. For example, flg22 peptides, a highly conserved region at the N-terminal of bacterial PAMP flagellin, can induce plant immunity and act on SA, MAPK, and other signal pathways, thus having an important impact on plant disease resistance, stomatal movement, growth, and development (Zipfel et al., 2004). The SCRE^11–179^ from *Ustilaginoidea virens* effector SCRE1 could suppress pattern-triggered rice immunity, and the ^45^CPARS^49^ motif has proven to be crucial for its suppression activity (Zhang et al., 2020).

As an important post-translational modification (PTM), protein ubiquitination is widely involved in various life activities, such as plant growth and development, biological and abiotic stress response, cycle regulation, and signal transduction by mediating the degradation of specific proteins (Stone et al., 2005; Liu and Stone, 2011). The ubiquitination system is a complex reaction process including three enzymes, namely E1 ubiquitin-activating enzyme, E2 ubiquitin-binding enzyme, and E3 ubiquitin ligase (Marino et al., 2012). E3 ubiquitin ligase can specifically recognize target proteins and has been proved important in multiple stages of plant immunity (Nagels et al., 2016). E3 ubiquitin ligases are divided into HECT, U-box, RING, and CRLs4 according to their domains and binding modes with substrates. The RING domain is a Cys-rich region made up of 40∼60 amino acids and contains eight spatially conserved Cys and His residues as metal ligands to chelate two zinc ions to realize the transfer of ubiquitin to the target protein (Marino et al., 2012). Many RING-type E3 ligase proteins have been identified as plant immune modulators. For example, RING-H2 finger protein EL5 in response to rice n-acetylchitosan oligosaccharide elicitor and ATL gene family members of RING zinc finger E3 ubiquitin ligase activated by elicitor treatment in *Arabidopsis* have been proven to play essential roles in plant defense in response to pathogen infection (Durrant et al., 2000). Constitutive expression of ATL2 in *Arabidopsis* induces defense-related gene expression (Serrano and Guzmán, 2004). In pepper, encoding RING-type protein CaRING1 is an active E3 ligase, which is necessary for hypersensitive response (HR) and resistance response of pepper to the infection of avirulent *Xanthomonas campestris* pv *vesicatoria* (Lee et al., 2011).

In this study, the target protein of BxCDP1 was identified to explore the function of BxCDP1 in host pine by yeast two-hybrid screening assay and co-immunoprecipitation (Co-IP). In order to develop potential immune inducers of pines against *B. xylophilus*, the key peptide regions of the BxCDP1-triggered plant immunity were identified and the roles of these peptides in promoting the defense ability of the host were also explored. Moreover, the content of superoxide and H_2_O_2_ by BxCDP1 triggered in *N. benthamiana* was detected by the NBT and DAB staining, respectively. The callose deposits were also detected in *N. benthamiana* to further confirm that the plant immunity was stimulated by BxCDP1.

## Materials and methods

### Nematodes and plant materials

*Botrytis cinerea* was cultured on potato dextrose agar (PDA) plates at 25°C for 7 days. Then, AMA3, a *B. xylophilus* highly virulent strain, was transferred into the *B. cinerea* plates under the same culture conditions above. The nematodes were extracted using the Baermann funnel technique (Qiu et al., 2013). *N. benthamiana* was grown in a glasshouse at 25°C with a relative humidity of 60% under 16:8-h light:dark conditions, and the leaves of 4–5-week-old *N. benthamiana* plants were used in the infiltration assay. The 3-year-old *P. thunbergii* seedlings were cultivated at temperatures ranging from 28 to 32°C with the relative humidity ranging from 65 to 75%.

### Plasmid constructs

BxCDP1 and its 19 deletion mutants were cloned using *B. xylophilus* cDNA. These amplified fragments of BxCDP1 and its 19 deletion mutants were ligated into pBINRFP (pCAM1300-RFP) using the Clone Express II One Step Cloning Kit (Vazyme, Nanjing, China). The amplified fragments of BxCDP1 were also ligated into PVX (pGR107-3*HA). The interacting protein PtRHF1 of BxCDP1 was cloned using *Pinus thunbergii* cDNA. These amplified fragments of PtRHF1 were also ligated into pBINGFP [a plasmid containing green fluorescent protein (GFP)]. Individual colonies of each construct were examined by using PCR, and the positive clones were verified by sequencing. The primers we used are listed in Supplementary Table 1.

### *Agrobacterium tumefaciens* infiltration assays

The *A. tumefaciens* infiltration assays were performed according to previous operating instructions (Hu et al., 2019). Briefly, constructs were transformed into *A. tumefaciens* strain GV3101 by electroporation, and the cells were cultured on Luria Bertani (LB) agar plates with kanamycin and rifampicin. After culturing at 30°C with a rotation speed of 200 rpm for 12 h, the recombinant *A. tumefaciens* cells were resuspended in a wash buffer (10 mM MgCl_2_ and MES, and 100 μM acetosyringone, pH 5.6) and diluted to a final optical density (OD) at 600 nm of 0.4. The *A. tumefaciens* suspensions were infiltrated into the *N. benthamiana* leaves using a needleless syringe. After 7 days, the symptom development of infiltration points was observed and photographed. In the *A. tumefaciens* infiltration experiment of screening the key amino acid regions of BxCDP1, the empty vector pBINRFP was used as a control. The pBINGFP was used as the negative control when protein interaction verification was performed in *N. benthamiana*.

### Detection of superoxide, H_2_O_2_ content, and callose deposits triggered by BxCDP1 in *Nicotiana benthamiana*

Expression and purification of the recombinant BxCDP1 and GFP were performed as described (Hu et al., 2020; Wen et al., 2021). Briefly, the ORFs for BxCDP1 and GFP were amplified using specific primers (Supplementary Table 1). The purified PCR products were inserted into the linearized vector pPICZαA (containing a 6 × His tag). After transforming into *Escherichia coli* TOP10, validating the transformants, and sequencing, the pPICZαA vector containing BxCDP1-6 × His and GFP-6 × His was transformed into the *Pichia pastoris* KM71H (Invitrogen). Positive clones were grown in a yeast extract–peptone–dextrose (YPD) medium containing 100 μg/ml of zeocin at 30°C for 3 days. The cultures were centrifuged to procure the supernatant containing BxCDP1-6 × His and GFP-6 × His after shaking in the BMGY (buffered glycerol complex medium) and BMMY (buffered methanol complex medium) media for 4 days. The expressed proteins were validated by SDS-PAGE. Purification of the recombinant protein BxCDP1-6 × His and GFP-6 × His from the culture supernatant was performed by affinity chromatography using Ni-NTA Superflow resin (Qiagen). To further detect the induction of superoxide, H_2_O_2_, and callose deposits, 1 μM purified BxCDP1 protein was infiltrated into leaves of *N. benthamiana*, and at 3 h after injection, the infiltrated into *N. benthamiana* leaves were stained using sodium azide, 3,3′-diaminobenzidine (DAB), or aniline blue as described (Dong et al., 2011). Meanwhile, the infiltrated leaves of *N. benthamiana* with 1 μM purified BxCDP1 protein were immersed in 40 ml of decolorizing solution (absolute ethanol:water:lactic acid:glycerin:phenol = 8:1:1:1:1) after injection of 3 h, and soaked in vacuum for 30 min. The samples were bathed in a 60°C water bath for 30 min to remove chlorophyll. After washing with water, the leaves without chlorophyll were immersed in 0.01% (w/v) aniline blue staining solution for 2–4 h under dark conditions. Then, the deposition of callose was observed under a laser confocal microscope (excitation light wavelength 377 nm and emission light wavelength 447 nm). The *N. benthamiana* leaves infiltrated with purified GFP protein were used as a control. The staining area of DAB or NBT and the number of callose deposition in *N. benthamiana* were quantified using ImageJ software, respectively. The staining area of DAB or NBT and the number of callose deposition by purified GFP protein treated were both set as 1. The relative staining area of DAB or NBT and the relative number of callose deposits by purified BxCDP1 protein treated were calculated, respectively. The experiment was performed three times, and three different plants with three infiltrated leaves were used for each assay.

### Construction of yeast library

About 3000 *B. xylophilus* virulent strains of AMA3 were inoculated to 3-year-old *P. thunbergii*. The stem segments of *P. thunbergii* about 3 cm near the inoculation site were cut into small segments at 12 h, 5 days, and 10 days after *B. xylophilus* infection. The samples were then mixed with the stem segments without *B. xylophilus* inoculation (0 h), followed by wrapping with tin foil and quick-frozen by using liquid nitrogen, and sent to Shanghai Ouyi Biomedical Co., Ltd. for pine yeast library construction.

### Protein interaction analysis

BxCDP1 (without signal peptide) was cloned into the pGBKT7 bait vector and then transformed into yeast strain Y2H Gold. The *B. xylophilus*-infected pine cDNA library was screened following the Clontech protocols. Then, pGBKT7::BxCDP1 and potential interaction target proteins were performed in a yeast two-hybrid assay. In the Co-IP assay, BxCDP1 and PtRHF1 were cloned into PVX and pBINGFP and introduced into *A. tumefaciens* GV3101. The *A. tumefaciens* suspensions of PVX::BxCDP1, pBINGFP::PtRHF1, and their mixed bacterial suspensions were infiltrated into *N. benthamiana*. At 48 h after infiltration, the proteins were extracted and Co-IP assays were performed as described previously (Li et al., 2018).

### Peptide synthesis

The sequences of three deletion mutants of BxCDP1 (namely M9, M15, and M16), which triggered cell death in *N. benthamiana*, were synthesized by GenScript Biotech Corporation (Nanjing, China). The sequence of a deletion mutant of BxCDP1 that did not trigger cell death was also synthesized as a control (namely M14).

### Protein extraction and western blotting

Agroinfiltrated *N. benthamiana* leaves were collected at 48-h post-inoculation (hpi). Total protein extraction and immunoblotting were performed according to a previous report (Yin et al., 2013). In brief, total proteins were separated by 12% SDS-PAGE and transferred to a polyvinylidene fluoride (PVDF) membrane (Bio-Rad, Hercules, CA, United States). The membranes were blocked with 5% (w/v) non-fat dry milk for 1 h at room temperature and then they were washed three times with PBS containing 0.1% Tween-20. Transient protein expression was assessed by incubating the membrane with a 1:5000 dilution of a primary mouse anti-HA antibody (Abmart, Berkeley Heights, NJ, United States) or an anti-RFP antibody (Abcam, Cambridge, United Kingdom) or an anti-GFP antibody (Abmart, Berkeley Heights, NJ, United States), followed by incubation with a goat anti-mouse secondary antibody at a 1:10,000 dilution (IRDye 800, 926-32210; LI-COR Biosciences, Lincoln, NE, United States). The four deletion mutants of BxCDP1 (M9, M15, M16, and M14) were analyzed by incubating the membrane with a 1:5000 dilution of a primary rabbit anti-RFP polyclonal antibody, followed by incubation with a peroxidase-conjugated goat anti-rabbit IgG secondary antibody at a 1:10,000 dilution (Zhongshan Bio-technique, Beijing, China). The results of protein expressions were observed by using an Odyssey^®^ LI-COR imaging system. The Ponceau S staining was used to confirm the protein loading.

### Inoculation assay

In the inoculation assay, we cut a small wound at the stem of the 3-year-old *P. thunbergii* seedling about 20 cm from the ground with a sterile scalpel, and the wound was as deep as the xylem of the pine tree. Then, the sealing film was wrapped around the wound to make a funnel, and 1 mL of synthesized peptides (M9, M15, and M16) and M14 (final concentration 50 μg/ml) (Xu et al., 2013) were added to the funnels. At 4 hpi, approximately 1,500 nematodes (a mixture of juveniles and adults) were inoculated into those previous funnels containing synthesized peptides, respectively. The morbidity degree of the *P. thunbergii* seedlings was classified into five different grades according to the previous study (Yu et al., 2012): 0, all needles of pines are green; I, a quarter of needles have turned yellow; II, half of the needles have turned yellow or brown; III, three-fourths of the needles have turned brown; and IV, the whole seedling withered. The formulas (Rempel and Hall, 1996) for calculating the infection ratio and disease severity index (DSI) of pine seedlings are as follows:


$$\text{Infection rate (\%)}=\frac{\text{Total number of infected plants}}{\text{Total number of plants}}\times 100$$

$$\text{Disease severity index (DSI)}=\frac{\Sigma \,\text{the number of infected plants}\times \text{symptom degree}}{\text{Total number of plants}\,\times \text{the highest symptom degree}}\times 100$$

The infection levels of *B. xylophilus* were detected, and the infection ratio and DSI *P. thunbergii* seedlings were calculated. The infection assay of *B. xylophilus* was performed three times and a total of 18 individual *P. thunbergii* seedlings for each treatment were used. In addition, some 3-year-old *P. thunbergii* seedlings were inoculated with *B. xylophilus* for 2.5 h, 6 h, 12 h, 24 h, and 3 days, respectively. Some 3-year-old *P. thunbergii* seedlings were, respectively, inoculated with 1 mL of purified BxCDP1 and GFP proteins (with a final concentration of 50 μg/ml) (Xu et al., 2013) as described above. Briefly, 1 mL of purified BxCDP1 and GFP protein solutions was added to the funnels made of sealing film. Subsequently, the top of the funnel was sealed with another sealing film to prevent the rapid volatilization of the protein solutions.

### Total RNA extraction and cDNA synthesis

The total RNA from each sample of *N. benthamiana* and *P. thunbergii* was separately extracted using the Plant Total RNA Kit (ZP405) (Zoman Biotech, Beijing, China), containing DNA digestive enzymes. The concentration of the total RNA was detected using an Ultraspec TM 2100 Pro UV/visible spectrophotometer, and the integrity of the total RNA was detected using 1% agarose gels.

The total RNA of each sample was reverse transcribed into cDNA using HiScript II Q RT SuperMix for qPCR (+gDNA wiper) (Vazyme, Nanjing, China) according to the manufacturer’s instructions. The protocol consists of two steps. First, gDNA of the total RNA was removed when the reaction mixture was incubated at 42°C for 2 min. Then, reverse transcription of total RNA was performed by following the procedure at 50°C for 15 min and 85°C for 5 s.

### RT-qPCR assays

RT-qPCR assays were carried out using ChamQ SYBR qPCR Master Mix (Low ROX Premixed) (Vazyme, Nanjing, China) according to the manufacturer’s instructions. The RT-qPCR assays were kept at 95°C for 30 s and at 40 cycles of 95°C for 10 s, 60°C for 34 s, and 95°C for 15 s. Three 300 nM synthesized peptides (M9, M15, and M16) were, respectively, infiltrated into *N. benthamiana* for 3 h; then, the relative expressions of three PTI marker genes (*NbAcre31*, *NbPTI5*, and *NbCyp71D20*) were detected to evaluate the effects of the three peptides on them. *N. benthamiana* infiltrated with 300 nM synthesized peptide M14 was used as a control. Each assay was replicated three times, and the *NbEF1*α of *N. benthamiana* was used as a constitutively expressed endogenous control gene (Ma et al., 2015).

Relative transcript levels of PR genes (*PtPR-3*, *PtPR-4*, and *PtPR-5*) of 3-year-old *P. thunbergii* seedlings were detected after inoculating *B. xylophilus* with 50 μg/mL of synthesized peptides M9, M15, and M16, and the *P. thunbergii* seedlings inoculated with 1500 *B. xylophilus* (a mixture of juveniles and adults) with M14 were used as the control. The relative expressions of *PtRHF1* were measured when *P. thunbergii* were infected with *B. xylophilus* for 2.5 h, 6 h, 12 h, 24 h, and 3 days, respectively. Moreover, the relative expressions of *PtRHF1* were also measured when *P. thunbergii* were inoculated with BxCDP1 and GFP purified proteins. The assay was performed three times, and the *PtEF1*α of *P. thunbergii* was used as an endogenous control gene (Hirao et al., 2012).

The relative expression levels of genes above were analyzed and calculated using the ABI Prism 7500 software (Applied Biosystems, Foster City, CA, United States) and the 2^–ΔΔ*Ct*^ method, respectively. The RT-qPCR was conducted with three biological replicates and three technical replicates. All primer sequences are provided in Supplementary Table 1.

### Statistical analysis

All experiments were repeated at least three times with similar results. The statistical significance was determined by one-way ANOVA followed by Duncan’s multiple range test using SPSS ver. 19 (SPSS Japan Inc., Tokyo, Japan).

## Results

### BxCDP1 induces reactive oxygen species accumulation and callose deposits

**FIGURE 1:**
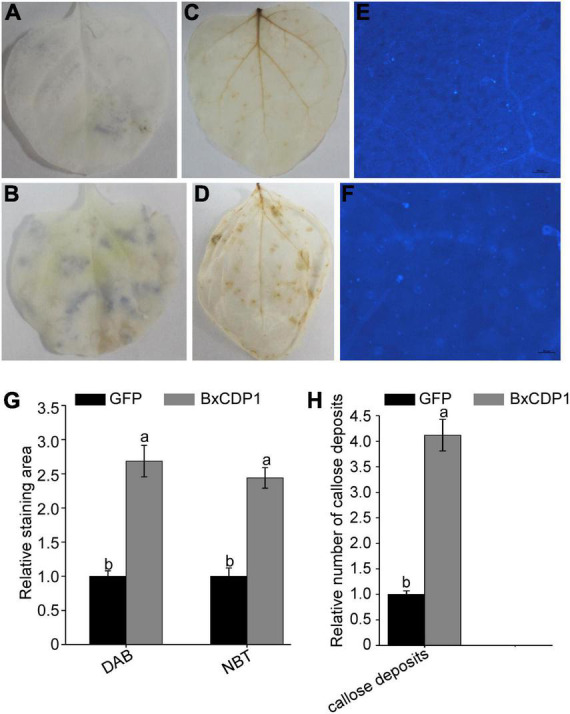
BxCDP1 induces the accumulation of superoxide and hydrogen peroxide and callose deposits in *Nicotiana benthamiana*. Representative *N. benthamiana* leaves stained using sodium azide after 3 h inoculation with purified GFP protein **(A)** and BxCDP1 protein **(B)**. Representative *N. benthamiana* leaves stained using 3,3’-diaminobenzidine (DAB) after 3 h inoculation with purified GFP protein **(C)** and BxCDP1 protein **(D)**. Representative *N. benthamiana* leaves stained using aniline blue after 3 h inoculation with purified GFP protein **(E)** and BxCDP1 protein **(F)**. These infiltration assays were, respectively, performed three times, and three different plants with three inoculated leaves were used in each assay. **(G)** Quantification using ImageJ software. The relative staining area of DAB or NBT by purified BxCDP1 protein treated in *N. benthamiana* was calculated. The staining area of DAB or NBT by GFP treated was set as 1. **(H)** The number of callose deposits per microscopic field was quantitated using ImageJ software. The relative number of callose deposits by purified BxCDP1 protein treated in *N. benthamiana* was counted. The number of callose deposits by GFP treated was set as 1. Data are the means, and the error bars represent ± SD from three biological replicates. Different letters on top of the bars indicate statistically significant differences (*p* < 0.05, *t*-test) as measured by Duncan’s multiple range test.

Nitroblue tetrazolium (NBT) can react with O^2–^ produced in plants to form a dark blue insoluble complex (Doke, 1983). Under dark conditions, DAB can react with hydrogen peroxide (H_2_O_2_) to produce dark brown precipitates visible to the naked eye (Thordal-Christensen et al., 1997). To determine whether BxCDP1 can induce ROS accumulation, the leaves of *N. benthamiana* injected with BxCDP1 and GFP pure protein were treated with NBT and DAB, respectively. The results showed that there were more dark blue areas and more dark brown sediments on leaves injected with purified BxCDP1 protein (Figures 1A,B) than those injected with purified GFP protein in the *N. benthamiana* (Figures 1C,D), respectively. The staining area of DAB or NBT by the former treated was calculated, which was compared with that of the latter treated. It showed that the relative staining area of DAB or NBT by BxCDP1 treated was both significantly bigger than GFP treated, which showed that the production of superoxide and H_2_O_2_ by BxCDP1 triggered was significantly higher than the GFP triggered (Figure 1G). These results indicated that BxCDP1 could induce ROS accumulation.

Similarly, more callose was accumulated in *N. benthamiana* leaves injected with purified BxCDP1 protein than that injected with GFP (Figures 1E,F). The relative number of callose deposits by BxCDP1 triggered was also significantly more than the GFP triggered (Figure 1H). The result further verified that BxCDP1 could induce plant immunity.

### BxCDP1 interacts with PtRHF1

**FIGURE 2:**
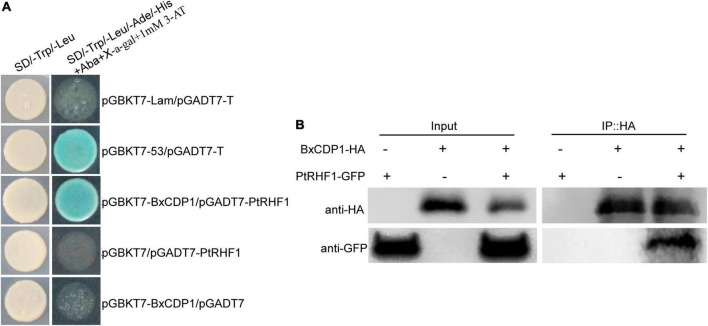
BxCDP1 interacts with *Pinus thunbergii* protein PtRHF1. **(A)** BxCDP1 interacts with PtRHF1 in yeast. Yeast strain Y2H Gold co-carrying pGBKT7-BxCDP1 and pGADT7-PtRHF1 was grown on SD/-Trp/-Leu and the selective medium SD/-Trp/-Leu/-Ade/-His+X-α-gal+AbA+1mM 3-AT. **(B)** BxCDP1 interacts with PtRHF1 *in vivo*. Co-immunoprecipitation (Co-IP) was performed on extracts of *Nicotiana benthamiana* leaves expressing both BxCDP1-HA and PtRHF1-GFP. BxCDP1-HA and PtRHF1-GFP were detected using anti-HA and anti-GFP antibodies by Western blot. The immune complexes were pulled down using anti-HA agarose beads.

To explore the avirulence function of BxCDP1 in host pine, a yeast two-hybrid screening assay was performed to identify potential host targets of BxCDP1. The result showed that BxCDP1 was self-activated in yeast (Supplementary Figure 1) and SD/-Trp/-Leu/-His/-Ade/+X-α-Gal+AbA plates with 1 mM 3-AT could effectively inhibit BxCDP1 self-activation (Supplementary Figure 2). The bait plasmid PGBKT7::BxCDP1 was co-transferred with yeast library plasmids of pine into yeast competent cell Y2H. Sixty-six positive transformants were screened on SD/-Trp/-Leu/-His/-Ade/+X-α-Gal+AbA plates with a 1-mM 3-AT plate. Then, the 66 positive transformants were sequenced and BLAST compared to a reference genome of *Pinus taeda* (Neale et al., 2014), and we found that most of them were not annotated (Supplementary Table 2). Among them, a RING-H2 finger protein 1 (RHF1) contains a RING-H2 domain, and some proteins containing this domain have been proved to participate in the immune response triggered by PAMP, the plant MAPK signal pathway, and apoptosis (Sinha et al., 2011; Park et al., 2012). At the same time, BxCDP1 proved to be a PAMP of *B. xylophilus* (Hu et al., 2020), so the PtRHF1, in which we were most interested in, was selected for subsequent one-to-one verification. The complete coding sequence of RHF1 from *P. thunbergii* was acquired according to the specific primer of *P. taeda* (Supplementary Table 1). The interaction between the full-length sequences of BxCDP1 and PtRHF1 was confirmed by one-to-one validation in yeast (Figure 2A). As expected, Co-IP experiments proved that BxCDP1 interacted with PtRHF1 in plants when they were co-expressed in *N. benthamiana* (Figure 2B). The results showed that PtRHF1, indeed, interacts with BxCDP1.

**FIGURE 3:**
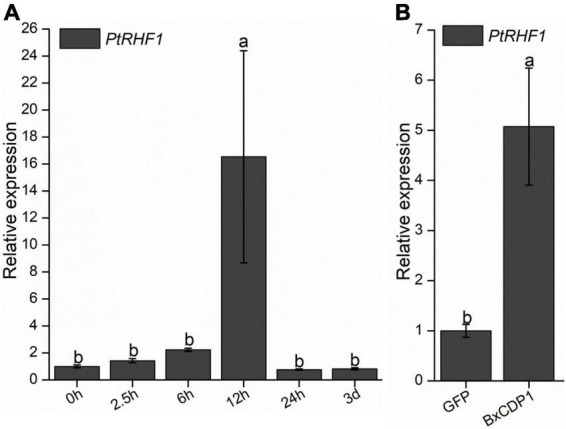
Relative transcript levels of *PtRHF1* in *Pinus thunbergii*. **(A)** The *PtRHF1* was upregulated at the early stages of *Bursaphelenchus xylophilus* infection. The *P. thunbergii* inoculated without *B. xylophilus* was used as a control. **(B)** The relative expression of *PtRHF1* was induced when purified BxCDP1 protein was inoculated into *P. thunbergii.* The *P. thunbergii* inoculated with purified GFP protein was used as a control. Data are the means, and the error bars represent ± SD from three biological replicates. Different letters on top of the bars indicate statistically significant differences (*p* < 0.05, *t*-test) as measured by Duncan’s multiple range test.

To investigate whether PtRHF1 is involved in the interaction between pine and *B. xylophilus*, the expression of *PtRHF1* was detected when *P. thunbergii* was inoculated with *B. xylophilus* for 0 h, 2.5 h, 6 h, 12 h, 24 h, and 3 days by RT-qPCR, respectively. The result showed that the expression of *PtRHF1* was upregulated at 2.5 h after *B. xylophilus* infection and reached the peak at 12 h, thus showing a downward trend (Figure 3A). These results suggested that the expression of *PtRHF1* was induced by the early infection of *B. xylophilus* and might be involved in the plant immune response.

In order to explore the relationship between BxCDP1 and PtRHF1, purified BxCDP1 protein was inoculated in 3-year-old *P. thunbergii* seedlings, and 6 h later, the relative expression levels of *PtRHF1* were detected in pine seedlings. The results showed that the relative expression level of *PtRHF1* in *P. thunbergii* inoculated with purified BxCDP1 protein was significantly higher than that of the control (Figure 3B), indicating that BxCDP1 can induce the upregulation of *PtRHF1*, suggesting that BxCDP1 may stimulate the defense response of *P. thunbergii* by inducing the upregulation of *PtRHF1*.

### The 56–86aa, 107–136aa, and 153–173aa are the key areas where BxCDP1 triggers cell death in *Nicotiana benthamiana*

**FIGURE 4:**
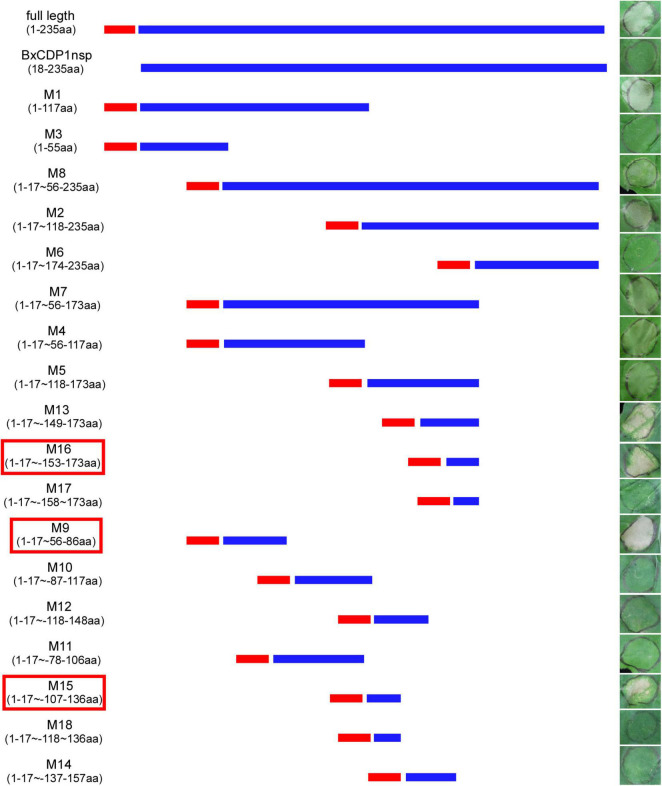
Analysis of domain by constructing deletion mutants of BxCDP1. The representative *Nicotiana benthamiana* leaves at 7 days after agroinfiltration carrying BxCDP1 and its 19 deletion mutants. M9, M15, and M16 were the shortest regions that could trigger cell death in *N. benthamiana.* The infiltration assay was performed three times and three different *N. benthamiana* with three inoculated leaves were used in each assay.

**FIGURE 5:**
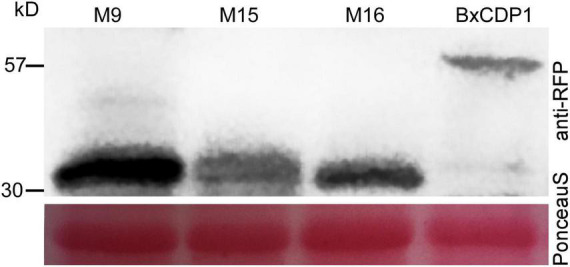
Western blot analysis of proteins from *Nicotiana benthamiana* leaves transiently expressing target proteins fused with anti-red fluorescent protein (anti-RFP) tags. Protein loading is shown by Ponceau S staining of RuBisCO.

In order to determine the key amino acid region of BxCDP1 triggering cell death in *N. benthamiana*, 19 deletion mutants of BxCDP1 were constructed and finally transiently expressed these deletion mutants into *N. benthamiana*. It was found that the mutant lacking the N-terminal signal peptide could not cause cell death in *N. benthamiana*, indicating that the deletion of N-terminal signal peptide affected its function of causing death in *N. benthamiana*. However, 10 mutants from the remaining 18 BxCDP1 deletion mutants adding the N-terminal signal peptide could cause cell death in *N. benthamiana*, and three of them, namely M9, M15, and M16 (56–86aa, 107–136aa, and 153–173aa), were in the shortest region that could trigger cell death in *N. benthamiana* (Figure 4). The expression of the three deletion mutants of BxCDP1 was validated by Western blot analysis (Figure 5). In addition, we found that the degree of cell death induced by a deletion mutant M8 (including regions covered by M9, M15, and M16) was lower than that induced by M9, M15, and M16. We speculated that this could be due to two reasons. First, the protein has a spatial structure. In M8, the regions covered by M9, M15, and M16 may be partially wrapped in space, resulting in a lower degree of cell death. Second, the expression of different proteins mediated by *A. tumefaciens* may be different. That is to say, the expression of M8 mediated by *A. tumefaciens* may be lower than that of M9, M15, and M16 mediated by *A. tumefaciens*, resulting in a lower degree of cell death.

### M9, M15, and M16 induce immune responses in *Nicotiana benthamiana*

**FIGURE 6:**
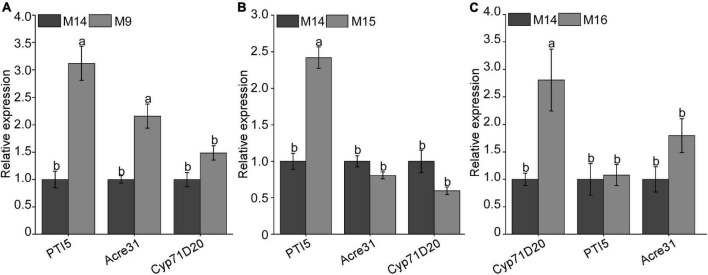
Detection of the relative expression of PAMP-triggered immunity (PTI) marker gene after injecting with three key peptides of BxCDP1 in *Nicotiana benthamiana.* **(A–C)** Transcriptional upregulation of *N. benthamiana* PTI marker genes triggered by 300 nM synthesized peptides M9, M15, and M16 in *N. benthamiana*. The *N. benthamiana* leaves infiltrated with synthesized peptide M14 were used as a control. The assay was conducted three times, and three different plants with three inoculated leaves were used in each assay. Data are the means, and the error bars represent ± SD from three biological replicates. Different letters on top of the bars indicate statistically significant differences (*p* < 0.05, *t*-test) as measured by Duncan’s multiple range test.

In the previous study, BxCDP1 could significantly induce the expression of PTI marker genes *NbAcre31*, *NbPTI5*, and *NbCyp71D20* (Hu et al., 2020). To determine whether the three regions (M9, M15, and M16) that triggered cell death can also induce immune responses in *N. benthamiana*, the sequences of M9, M15, and M16 and control M14 were synthesized. The 300 nM synthesized peptides M9, M15, and M16 and control M14 were infiltrated into leaves of *N. benthamiana*. The result showed that, compared with control M14, M9 could significantly induce the expressions of *NbAcre31* and *NbPTI5* (Figure 6A), M15 could significantly induce the expression of *NbPTI5* (Figure 6B), and M16 could significantly induce the expression of *NbCyp71D20* (Figure 6C). The result indicated that M9, M15, and M16 can significantly induce immune responses in *N. benthamiana.*

### M9 and M16 induce pathogenesis-related marker genes expression of *Pinus thunbergii*

**FIGURE 7:**
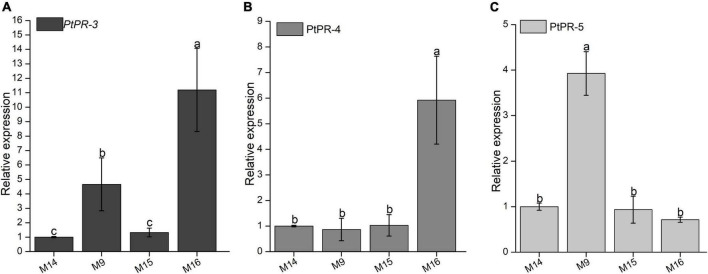
Relative transcript levels of pathogenesis-related (PR) genes in *Pinus thunbergii* inoculated with three synthesized peptides and *Bursaphelenchus xylophilus*. **(A)** The relative expression of *PtPR-3* was detected by RT-qPCR. **(B)** The relative expression of *PtPR-4* was detected by RT-qPCR. **(C)** The relative expression of *PtPR-5* was detected by RT-qPCR. The *P. thunbergii* seedlings were treated with three synthesized peptides M9, M15, and M16 for 4 h and then inoculated with *B. xylophilus*. After 6 h, 2 cm stems in the length of *P. thunbergii* seedlings were used for RNA extraction, and the relative expressions of *PtPR-3*, *PtPR-4*, and *PtPR-5* were detected. The *P. thunbergii* seedlings inoculated with *B. xylophilus* after inoculating with synthesized peptide M14 were used as the control. The inoculation assay was repeated three times, and in each assay, three different seedlings for each treatment were used. Data are the means, and the error bars represent ± SD from three independent experiments. Different letters on top of the bars indicate statistically significant differences (*p* < 0.05, *t*-test) as measured by Duncan’s multiple range test.

Our previous study found that BxCDP1 can induce the expression of pathogenesis-related marker genes *PtPR-3*, *PtPR-4*, and *PtPR-5* of *P. thunbergii*, and *P. thunbergii* could sense the presence of xenobiotics and stimulate defense response (Hu et al., 2020). Therefore, in order to explore whether the identified peptides that can trigger cell death and immunity in *N. benthamiana* also have the ability to induce the expression of pathogenesis-related marker genes in *P. thunbergii*, M9, M15, M16, and control M14 were inoculated with 3-year-old *P. thunbergii* for 4 h, and then, about 1500 *B. xylophilus* strains were inoculated. The relative expressions of *PtPR-3*, *PtPR-4*, and *PtPR-5* were detected by RT-qPCR. Compared with the expression levels in M15 and control M14-treated *P. thunbergii*, *PtPR-3* was significantly upregulated both in the M9-treated and the M16-treated *P. thunbergii* (Figure 7A), *PtPR-4* was significantly upregulated in the M16-treated samples (Figure 7B), and *PtPR-5* was significantly upregulated in the M9-treated samples (Figure 7C).

### M9 and M16 enhance the resistance of *Pinus thunbergii* to *Bursaphelenchus xylophilus*

**FIGURE 8:**
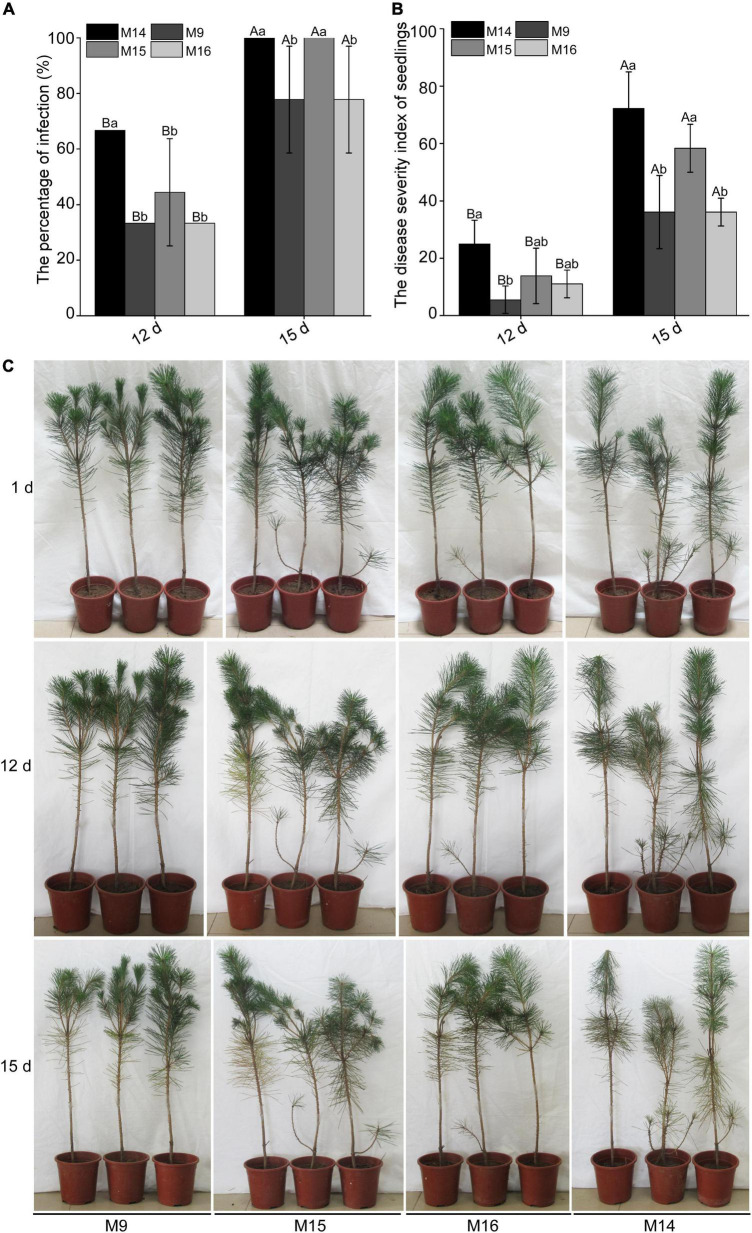
Peptides M9 and M16 of BxCDP1 can promote the resistance of *Pinus thunbergii* to *Bursaphelenchus xylophilus*. **(A)** The infection rates of *P. thunbergii* seedlings under four different treatments. **(B)** The disease severity index of *P. thunbergii* seedlings under four different treatments. The *P. thunbergii* seedlings were treated with three synthesized peptides M9, M15, and M16 for 4 h and then inoculated with 1500 *B. xylophilus*. The *P. thunbergii* seedlings inoculated with 1500 *B. xylophilus* after treating with synthesized peptide M14 for 4 h were used as control. Data are the means of three independent inoculation experiments and error bars (±SD) from three independent inoculation experiments. Different capital letters above the bars indicate significant differences between inoculation times in the same treatments (*p* < 0.05, *t*-test), as measured by Duncan’s multiple range test; different lowercase letters above the bars indicate significant differences between different treatments at the same inoculation time (*p* < 0.05, *t*-test), as measured by Duncan’s multiple range test. The infection experiment was repeated three times with three replicate *P. thunbergii* seedlings per treatment. **(C)** Representative photographs of *P. thunbergii* at 1 day, 12 days, and 15 days post-inoculation. The *B. xylophilus* infection assay was conducted three times.

M9 and M16 could stimulate the expression of pathogenesis-related marker genes. However, it is unclear whether they can improve the resistance of *P. thunbergii* to *B. xylophilus.* Thus, M9, M15, and M16 and control M14 were inoculated into *P. thunbergii* for 4 h, and approximately 1,500 mixed-life-stage highly virulent strains of *B. xylophilus* AMA3 were inoculated at the inoculation wound of *P. thunbergii*. The pines infected with *B. xylophilus* would show early symptoms such as yellowing or browning or wilting needles. The result showed that this early symptom occurred later in seedlings inoculated with M9 and M16, compared with the ones inoculated with M14 and M15, whether on the 12 dpi or 15 dpi (Figure 8C). Moreover, the infection ratio and disease severity index of *P. thunbergii* seedlings inoculated with M9 and M16 were also significantly lower than the seedlings inoculated with M15 and control M14 at both 12 dpi and 15 dpi (Figures 8A,B).

## Discussion

The burst of plant ROS, the upregulated expression of PR genes, and callose deposits are the indicators of plant immunity activation (Dodds and Rathjen, 2010). Our team identified a PAMP BxCDP1 in *B. xylophilus*, which can trigger cell death, and upregulated the expression of PTI marker genes and ROS outbreak in various plants, namely, *N. benthamiana* (Hu et al., 2020). In this study, the accumulation of superoxide, H_2_O_2_, and callose deposits in *N. benthamiana* treated with purified BxCDP1 protein was significantly higher than that in the control. It indicated that BxCDP1 promoted the outbreak of ROS by stimulating the production of more superoxide and H_2_O_2_ in *N. benthamiana* and callose deposits further verified that the immune response of *N. benthamiana* was activated by BxCDP1. These results indicated that BxCDP1 was a key molecular pattern to plant immunity activation.

Identifying the target proteins of PAMPs or effectors in the host can further reveal how PAMPs or effectors play a role in the infection of *B. xylophilus*. Many RING-type E3 ligase proteins have been identified as plant immune modulators. For example, the effector AvrPiz-t of *Magnaporthe oryzae* can be transported to rice cells and combined with the ubiquitin ligase APIP6 of rice RING E3 to make it unstable so as to inhibit the immunity induced by PAMP (Park et al., 2012). The expression of rice RING-type zinc finger protein OSRHC1 in *Arabidopsis* improves the resistance to highly toxic bacteria (Cheng et al., 2007). Moreover, the known homologous proteins containing the RING domain have been proven to be involved in ubiquitin-mediated protein hydrolysis, plant MAPK signal pathway, and apoptosis. Among them, plant MAPK and its signal transduction are considered to be important receptors and signal transduction processes involved in PAMPs signal transduction (Sinha et al., 2011). In this study, PtRHF1 was predicted to contain a RING-H2 domain and proved to be a host target protein interacting with PAMP BxCDP1 that activates plant PTI. Thus, we speculate that PtRHF1 may be a plant E3 ligase that participates in the ubiquitination process of BxCDP1 into plants so as to activate plant immunity. The relative expression of *PtRHF1* started to increase at 2.5 h and reached the peak at 12 h after *B. xylophilus* infection, indicating that PtRHF1 played an important role in the early stage of *B. xylophilus* infection. In addition, the expression of *PtRHF1* was significantly induced after purified BxCDP1 protein was inoculated into *P. thunbergii*. These results suggested that PtRHF1 was involved in the early immune response BxCDP1 triggered, which works by stimulating the defense response of *P. thunbergii* by inducing the upregulation of *PtRHF1*. However, because both the gene knockout and gene silencing system are not suitable for gymnosperm pine, we are restricted from further exploring the exact biological function of PtRHF1 in pine. Hence, in order to tackle the above speculation, multiple proofs of experiments need to be carried out in future. Next, we will further explore subcellular location and influence on nematodes (that can infect *N. benthamiana*, such as root-knot nematode) infection of PtRHF1 in *N. benthamiana* so as to indirectly reveal its function in the interaction between nematodes and pine trees. Moreover, a target protein PtACO1 of *B. xylophilus* effector BxSCD1 that can inhibit BxCDP1-triggered cell death and immunity was identified (Wen et al., 2021). Thus, whether there is a certain correlation between the interaction of BxCDP1 and PtRHF1 and the interaction of BxSCD1 and PtACO1, and the effect of their correlation on *B. xylophilus* infection need to be further studied.

At present, one of the common methods to screen and identify the key amino acid regions is to construct a protein deletion mutant and express it transiently in *N. benthamiana*. For example, the key amino acid regions of the effector SCRE1 of *U. virens* and the effectors PsAvh238 and PsAvh52 of *Phytophthora sojae* that inhibit plant immunity were identified through this method (Yang et al., 2017; Li et al., 2018). In this study, we successfully screened and identified three short amino acid regions of BxCDP1 named M9, M15, and M16 (31aa, 30aa, and 21aa, respectively), which can independently trigger cell death in *N. benthamiana*. Moreover, as the full length of BxCDP1, M9 and M16 could trigger the upregulated expression of PTI marker genes, indicating that they, as key regions of BxCDP1, indeed, triggered plant immunity. However, their ability to trigger the expression of the PTI marker gene was weaker than the full length of BxCDP1 (Hu et al., 2020). We speculated that the ability of BxCDP1 to trigger plant immunity is the superposition of the two regions.

BxCDP1 triggered the upregulated expressions of the host PR genes *PtPR-3*, *PtPR-4*, and *PtPR-5* and consequently improved the pine resistance to PWD (Hu et al., 2020). In this study, we also found that synthesized peptides M9 could stimulate the upregulated expression of *PtPR-3* and *PtPR-5*, and M16 could induce the upregulated expression of *PtPR-3* and *PtPR-4* in *P. thunbergii*. Meanwhile, pathogenicity tests showed that the early symptom of *P. thunbergii* seedlings inoculated with *B. xylophilus* after M9 and M16 occurred later than the control pines to some extent. Altogether, these results showed that both M9 and M16 could enhance the resistance of *P. thunbergii* seedlings to *B. xylophilus* by stimulating the expressions of host PR genes. Among all the synthesized peptides, M9 works the best in triggering cell death, inducing PTI Marker genes’ expression and improving pine resistance, followed by M16. In conclusion, both M9 and M16 have the potential to be developed and utilized as immune inducers against *B. xylophilus* in future.

## Data availability statement

The original contributions presented in this study are included in the article/Supplementary material, further inquiries can be directed to the corresponding author.

## Author contributions

X-QW and L-JH were the leading investigators of this research program and planned and designed the research. L-JH performed the majority of experiments with the help of T-YW, Y-JQ, LR, and YZ. X-QW and J-RY contributed reagents, materials, and analysis tools. L-JH analyzed the data and wrote the manuscript with suggestions from X-QW and T-YW. All authors commented on the manuscript before submission and approved the submitted version.
