# Supplementary material for: The key molecular pattern BxCDP1 of Bursaphelenchus xylophilus induces plant immunity and enhances plant defense response via two small peptide regions
Source: Front Plant Sci. 2022 Aug 3;13:937473. doi: 10.3389/fpls.2022.937473 (PMC9382027; doi:10.3389/fpls.2022.937473)
Supplement: Supplementary file 1 [file Data_Sheet_1.docx]

Supplementary Material

## Supplementary Figure S1


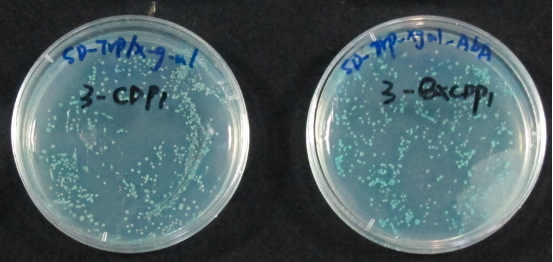


**Figure S1 Detection of self-activation ability of BxCDP1 in *Pichia pastoris.*** The yeast cells carrying pGBKT7:: BxCDP1 and pGADT7 could grow and turn blue on SD/-Trp/-Leu+X-α-Gal and SD/-Trp/-Leu+X-α-Gal+AbA plates.

## Supplementary Figure S2


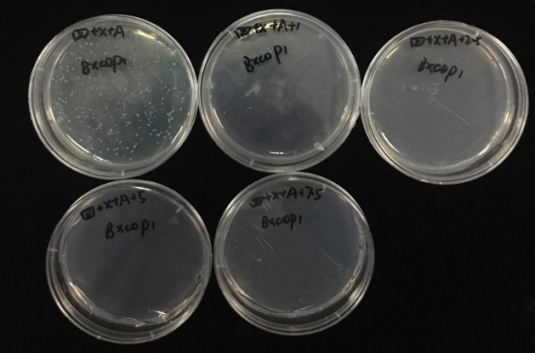


**Figure S2 Screening of 3-AT concentration inhibiting BxCDP1 self-activation on SD**/**-Trp/-Leu/-His/-Ade/+X-α-Gal+AbA plates.** The yeast cells carrying pGBKT7:: BxCDP1 and pGADT7 could grow on SD/-Trp/-Leu/-His/-Ade/+X-α-Gal+AbA plates, but they could not grow on SD/-Trp/-Leu/-His/-Ade/+X-α-Gal+AbA plates with 1mM 3-AT plates.

| **Table S1 List of primers used in this study** | | | |
| --- | --- | --- | --- |
| Gene name | Forward primer sequence | Reverse primer sequence | Purpose |
| BxCDP1 | ATAGCCGGTACCCCCGGGATGAAGTGTGTTGTGGTCCTCT | GGAGGAGGCCATCCCGGGCAATGATGGGAAGGTGACTGG | Plasmid constructs for BxCDP1 and its 19 deletion mutants |
|  |  |  |  |
| BxCDP1nsp | ATAGCCGGTACCCCCGGGATGTTCCCAGGCATTCCATCC | GGAGGAGGCCATCCCGGGCAATGATGGGAAGGTGACTGG |  |
|  |  |  |  |
| M1 | ATAGCCGGTACCCCCGGGATGAAGTGTGTTGTGGTCCTCT | GGAGGAGGCCATCCCGGGGAGGATCTTGTTCAGAAGCTC |  |
|  |  |  |  |
| M2 | CTTCTCCATGGCCCAAGGCGAATCTCTGAACCTCCCCAC | GGAGGAGGCCATCCCGGGCAATGATGGGAAGGTGACTGG |  |
|  |  |  |  |
| M3 | ATAGCCGGTACCCCCGGGATGAAGTGTGTTGTGGTCCTCT | GGAGGAGGCCATCCCGGGCTCCAAAAGTGGCTCTTGTG |  |
|  |  |  |  |
| M4 | CTTCTCCATGGCCCAAGGCGCTTTGGTTCAAATTCTCAACC | GGAGGAGGCCATCCCGGGGAGGATCTTGTTCAGAAGCTC |  |
|  |  |  |  |
| M5 | CTTCTCCATGGCCCAAGGCGAATCTCTGAACCTCCCCAC | GGAGGAGGCCATCCCGGGGTTGATGGTGGGCAGGTGC |  |
|  |  |  |  |
| M6 | CTTCTCCATGGCCCAAGGCCCTGAGGAAGTGACCGTCC | GGAGGAGGCCATCCCGGGCAATGATGGGAAGGTGACTGG |  |
| M7 | CTTCTCCATGGCCCAAGGCGCTTTGGTTCAAATTCTCAACC | GGAGGAGGCCATCCCGGGGTTGATGGTGGGCAGGTGC |  |
|  |  |  |  |
| M8 | CTTCTCCATGGCCCAAGGCGCTTTGGTTCAAATTCTCAACC | GGAGGAGGCCATCCCGGGCAATGATGGGAAGGTGACTGG |  |
|  |  |  |  |
| M9 | CTTCTCCATGGCCCAAGGCGCTTTGGTTCAAATTCTCAACC | GGAGGAGGCCATCCCGGGTTCAACTCCCAATTCGGGGAA |  |
|  |  |  |  |
| M10 | CTTCTCCATGGCCCAAGGCGGAGACCGAGTCAAGAGAGA | GGAGGAGGCCATCCCGGGGAGGATCTTGTTCAGAAGCTC |  |
|  |  |  |  |
| M11 | CTTCTCCATGGCCCAAGGCGTTACTTTCCCCGAATTGGGA | GGAGGAGGCCATCCCGGGCAATGGAGATGGTGGAAGGC |  |
|  |  |  |  |
| M12 | CTTCTCCATGGCCCAAGGCGAATCTCTGAACCTCCCCAC | GGAGGAGGCCATCCCGGGGGTGACTGGCTCGAAGGCA |  |
|  |  |  |  |
| M13 | CTTCTCCATGGCCCAAGGCGCTGAGCCCATTTTCGACGT | GGAGGAGGCCATCCCGGGGTTGATGGTGGGCAGGTGC |  |
|  |  |  |  |
| M14 | CTTCTCCATGGCCCAAGGCGGCCGTGTCAAGAGAGATGC | GGAGGAGGCCATCCCGGGGAGCAGAACGTCGAAAATGGG |  |
|  |  |  |  |
| M15 | CTTCTCCATGGCCCAAGGCGTCGTAATCATCGAGCTTCTG | GGAGGAGGCCATCCCGGGAGCATCAGAAGGGATGGTGAC |  |
| M16 | CTTCTCCATGGCCCAAGGCTTCGACGTTCTGCTCGACATC | GGAGGAGGCCATCCCGGGGTTGATGGTGGGCAGGTGC |  |
|  |  |  |  |
| M17 | CTTCTCCATGGCCCAAGGCGACATCCTGAACCAGATCTTG | GGAGGAGGCCATCCCGGGGTTGATGGTGGGCAGGTGC |  |
|  |  |  |  |
| M18 | CTTCTCCATGGCCCAAGGCGAATCTCTGAACCTCCCCAC | GGAGGAGGCCATCCCGGGAGCATCAGAAGGGATGGTGAC |  |
|  |  |  |  |
| PBINRFP vector | CGAATCTCAAGCAATCAAGCA | CTCCATGCGCACCTTGAAG |  |
|  |  |  |  |
| NbAcre31 | AATTCGGCCATCGTGATCTTGGTC | GAGAAACTGGGATTGCCTGAAGGA | RT-qPCR for PTI marker genes expression levels in *Nicotiana benthamiana* |
| NbPTI5 | CCTCCAAGTTTGAGCTCGGATAGT | CCAAGAAATTCTCCATGCACTCTGTC |  |
| NbCyp71D20 | GTTGACGCCATTGTTGAG | ATCTTCGCCTCCTAATGC |  |
| NbEF1α | GTATGCCTGGGTGCTTGAC | ACAGGGACAGTTCCAATACCA |  |
| PtPR-3 | ACCTACAGCGCCTTCATTGC | TGTGGTTTCATGCGACGTTT | RT-qPCR for pathogenesis-related genes expression levels in *Pinus thunbergii* |
| PtPR-4 | CCCCGTTACTGTCAATTGCAT | AAAGCGTGACGGTGCGTATT |  |
| PtPR-5 | GAACCAGTGCCCATACACAGTCT | CCTGCGGCAACGTTAAAAGTC |  |
| PtEF1α | AGATTGGAAATGGTTATGCCCCTGT | CCAGAACGCCTGTCAACCTTGGT |  |
| pGBKT7:: BxCDP1 | GCCATGGAGGCCGAATTCATGTTCCCAGGCATTCCATCC | ACGGATCCCCGGGAATTCCAATGATGGGAAGGTGACTGG | Plasmid constructs for yeast two-hybrid |
|  |  |  |  |
| pGADT7::PtRHF1 | ATGGAGGCCAGTGAATTCATGAAGAGCAGACGATTAGCG | CCCACCCGGGTGGAATTCAGCCGGCGCCCTGTGCTC |  |
|  |  |  |  |
| PBINGFP::PtRHF1 | GTACAAGGGTACCCCCGGGATGAAGAGCAGACGATTAGCG | AGGATCCGTCGACCCCGGGAGCCGGCGCCCTGTGCTC | Plasmid constructs for Co-IP verification |
| PVXHA::BxCDP1 | CTAGCATCGATTCCCGGGATGAAGTGTGTTGTGGTCCTCT | CTCTAGAGGATCCCCGGGCAATGATGGGAAGGTGACTGG |  |
| PtRHF1 | CTACGGAATCATCGAGCCCC | GTCGAGTGTGAGCGACTGAA | RT-qPCR for PtRHF1 expression level in *Pinus thunbergii* |
| PtEF1α | AGATTGGAAATGGTTATGCCCCTGT | CCAGAACGCCTGTCAACCTTGGT |  |

| **Table S2 Information of positive interactors of BxCDP1 in yeast two-hybrid** | | |
| --- | --- | --- |
| Gene name | Gene ID | Annotation |
| Pt-1 | TRINITY_DN159001_c1_g2 | Protein Sawadee Homeodomain Homolog 2 |
| Pt-2 | TRINITY_DN158191_c1_g3 | 40S ribosomal protein SA |
| Pt-3 | TRINITY_DN147302_c0_g1 | C2H2 finger domain transcription factor |
| Pt-5 | TRINITY_DN160057_c1_g5 | Unknown |
| Pt-6 | TRINITY_DN146204_c0_g1 | Homeobox-leucine zipper protein |
| Pt-13 | TRINITY_DN161668_c1_g4 | Peptidyl-prolyl cis-trans isomerase (putative cyclophilin) |
| Pt-18 | No found | Unknown |
| Pt-20 | No found | Unknown |
| Pt-21 | No found | Unknown |
| Pt-23 | TRINITY_DN144692_c0_g1 | Tetratricopeptide repeat protein 1 |
| Pt-25 | No found | Unknown |
| Pt-28 | No found | Unknown |
| Pt-30 | RINITY_DN144692_c0_g1 | Tetratricopeptide repeat protein 1 |
| Pt-33 | TRINITY_DN168439_c2_g2 | Glycine-rich RNA-binding protein |
| Pt-34 | TRINITY_DN158191_c1_g3 | 40S ribosomal protein SA |
| Pt-40 | TRINITY_DN161668_c1_g4 | Putative cyclophilin |
| Pt-41 | TRINITY_DN164214_c0_g1 | Ethylene Insensitive 3-like 1 protein |
| Pt-42 | TRINITY_DN161668_c1_g4 | Putative cyclophilin |
| Pt-46 | TRINITY_DN161668_c1_g4 | Putative cyclophilin |
| Pt-47 | TRINITY_DN154583_c2_g4 | Cysteine proteinase inhibitor 3 |
| Pt-50 | TRINITY_DN158067_c1_g1 | Homeobox protein knotted-1-like 3 |
| Pt-51 | TRINITY_DN168779_c4_g3 | Fructose-bisphosphate aldolase 8, cytosolic |
| Pt-55 | TRINITY_DN160510_c1_g3 | Unknown |
| Pt-58 | TRINITY_DN162403_c0_g1 | Unknown |
| Pt-60 | No found | Unknown |
| Pt-62 | TRINITY_DN154583_c2_g4 | Cysteine proteinase inhibitor 3 |
| Pt-63 | TRINITY_DN154598_c0_g2 | Transcription factor bHLH68 |
| Pt-65 | TRINITY_DN154583_c2_g4 | Cysteine proteinase inhibitor 3 |
| Pt-66 | TRINITY_DN148260_c0_g1 | Rho GTPase-activating protein 1 |
| Pt-67 | TRINITY_DN149266_c0_g3 | Peroxisome biogenesis protein 22 |
| Pt-68 | TRINITY_DN160510_c1_g3 | Unknown |
| Pt-71 | TRINITY_DN167705_c0_g5 | Unknown |
| Pt-78 | TRINITY_DN152927_c1_g1 | Histidine-containing phosphotransfer protein 1 |
| Pt-80 | TRINITY_DN154583_c2_g4 | Cysteine proteinase inhibitor 3 |
| Pt-86 | TRINITY_DN161762_c2_g1 | Probable nucleoredoxin 1-1 |
| Pt-88 | TRINITY_DN161476_c0_g1 | Unknown |
| Pt-94 | TRINITY_DN167705_c0_g5 | Unknown |
| Pt-95 | TRINITY_DN166917_c1_g3 | Unknown |
| Pt-98 | TRINITY_DN161668_c1_g4 | Putative cyclophilin |
| Pt-102 | TRINITY_DN154583_c2_g4 | Cysteine proteinase inhibitor 3 |
| Pt-110 | No found | Unknown |
| Pt-114 | TRINITY_DN161668_c1_g4 | Putative cyclophilin |
| Pt-117 | TRINITY_DN147086_c0_g1 | Acyl carrier protein 1, mitochondrial |
| Pt-118 | TRINITY_DN169011_c2_g1 | RING-H2 finger protein |
| Pt-120 | No found | Unknown |
| Pt-123 | TRINITY_DN159911_c0_g1 | NtPRp27-like protein |
| Pt-128 | No found | Unknown |
| Pt-136 | TRINITY_DN160427_c0_g2 | Unknown |
| Pt-146 | No found | Unknown |
| Pt-155 | TRINITY_DN149993_c0_g4 | Unknown |
| Pt-156 | No found | Unknown |
| Pt-160 | TRINITY_DN154583_c2_g4 | Cysteine proteinase inhibitor 3 |
| Pt-161 | No found | Unknown |
| Pt-163 | TRINITY_DN154583_c2_g4 | Cysteine proteinase inhibitor 3 |
| Pt-167 | TRINITY_DN168439_c2_g2 | Glycine-rich RNA-binding protein |
| Pt-168 | TRINITY_DN153112_c1_g3 | Unknown |
| Pt-171 | TRINITY_DN161668_c1_g4 | Putative cyclophilin |
| Pt-172 | TRINITY_DN160510_c1_g3 | Unknown |
| Pt-177 | TRINITY_DN142088_c0_g1 | Small heat shock protein C2 |
| Pt-186 | TRINITY_DN161695_c1_g1 | Mitochondrial import inner membrane translocase subunit TIM17-2 |
| Pt-188 | TRINITY_DN122432_c0_g1 | Unknown |
| Pt-189 | TRINITY_DN161668_c1_g4 | Putative cyclophilin |
| Pt-191 | TRINITY_DN169068_c3_g1 | Glucan endo-1,3-beta-glucosidase |
| Pt-193 | TRINITY_DN159072_c1_g1 | Desiccation-related protein |
| Pt-198 | No found | Unknown |
| Pt-200 | TRINITY_DN167251_c2_g1 | Chitinase 5 PR-3 |
